# Alcohol-Induced Cell Death in the Embryo

**Published:** 1997

**Authors:** Susan M. Smith, Susan M. Smith

**Affiliations:** Associate professor in the Department of Nutritional Sciences at the University of Wisconsin—Madison

**Keywords:** congenital facial anomaly, fetal alcohol syndrome, prenatal alcohol exposure, cytolysis, gestation, teratogens, neural cell, temporal context, retinoic acid, free radicals, oxygen, cell signaling, animal model, literature review

## Abstract

Exposure to alcohol during gestation can have profound consequences, but not all cells within the embryo are affected equally. Recent advances in molecular embryology have allowed an exploration of this variation. Much of this research has focused on the embryo’s vulnerability to the facial malformations characteristic of fetal alcohol syndrome. Studies using mice and chicks show that alcohol exposure at specific stages of early embryo development results in significant death among the cells destined to give rise to facial structures (i.e., cranial neural crest cells). This type of cell death is through activation of the cell’s own “self-destruct” machinery (i.e., apoptosis). Researchers have advanced several theories to explain how alcohol triggers apoptosis in the neural crest cells. These theories include deficiency in a type of vitamin A compound, retinoic acid; reduced levels of antioxidant compounds (i.e., free radical scavengers) that protect against damage from toxic oxygen molecules (i.e., free radicals); and interference with the cell’s normal internal communication pathways.

Alcohol is capable of directly inducing abnormalities during prenatal development that can lead to lifelong and profound disabilities (i.e., it is a teratogen^1^). In fact, prenatal exposure to alcohol—the most prevalent teratogen in Western society—is the most common known cause of mental retardation and neurobehavioral deficits. By conservative estimates, 12 percent of all patients in long-term institutionalized care have fetal alcohol syndrome (FAS) (see Stratton et al. 1996 for an extensive discussion of FAS). Although the simplest prevention strategy is for women to avoid alcohol consumption when pregnant or planning to conceive, one-half of all pregnancies are unplanned. By the time these pregnancies are confirmed, major embryonic events already have occurred. (For a brief overview of normal embryo development, see sidebar, p. 296–297.)

**Embryo Development**

The union of sperm and egg at fertilization sets into motion a remarkable series of events destined to culminate in a fully developed adult. As development proceeds toward this goal, cells divide, differentiate into specific types, and organize into body tissues and systems according to both an ancient general schematic for the species and the new person’s unique genetic heritage.

The first 8 weeks of this process (i.e., the embryo stage) are especially busy and are crucial in laying the foundation for future growth (see figure 1). From its tiny, one-celled beginning, a fertilized egg undergoes a process of rapid cell division (i.e., cleavage) and soon becomes a roughly spherical cluster of cells called a blastocyst. Approximately 4 days after fertilization takes place in the fallopian tube (i.e., oviduct), the blastocyst arrives in the uterus.^1^ Within a few more days, the blastocyst attaches to the wall of the uterus, where it will remain anchored until birth. By the time attachment (i.e., implantation) occurs, the rate of cell division has slowed somewhat, and the cells of the blastocyst have rearranged themselves into a hollow ball. A flat disk of cells inside the ball will become the embryo proper, while the outer cells will develop into placental tissues.

**Figure 1:**
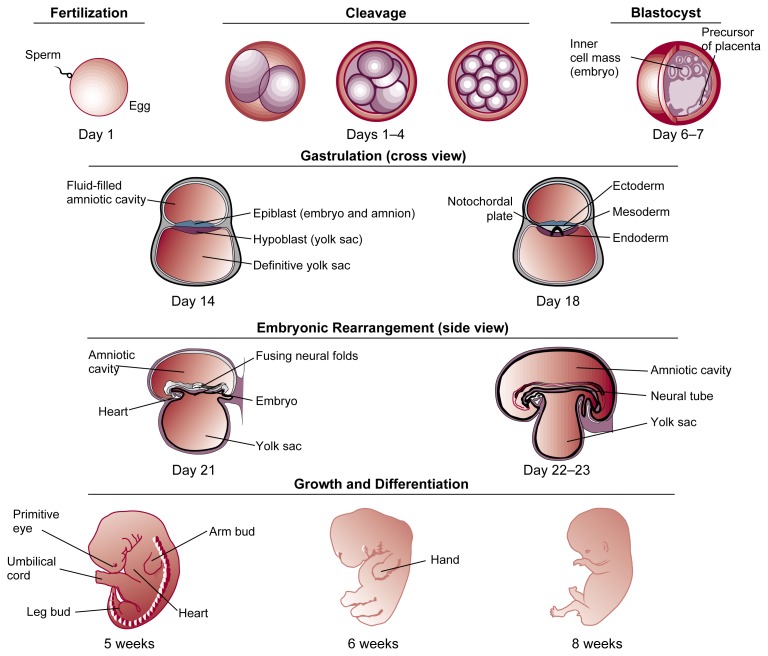
Key stages of embryo development. *(Note: Structures not drawn to scale.)*

Approximately 2 weeks after fertilization, further cell rearrangement (i.e., gastrulation) begins to establish a multilayered, vertebrate body plan in the embryo. Three distinct “founder regions” of cells (i.e., germ layers) form during gastrulation: the ectoderm, endoderm, and mesoderm. Unfolding genetic codes direct further development of each germ layer, transforming it from an initially undifferentiated set of cells to increasingly complex, genetically programmed tissue patterns that eventually become recognizable body structures. Thus, the skin and nervous system are formed from the outermost layer of cells (i.e., the ectoderm), while the lining of the digestive tract, respiratory tubes, and their associated organs derive from the innermost layer (i.e., the endoderm). Between these two layers, the mesoderm spreads outward and will generate all the organs between the ectodermal wall and the endodermal tissue, including the cardiovascular system, bones, muscles, and connective tissue.

The cells of the three germ layers interact with one another in an intricate sequence to coordinate the development of all the body structures. For example, a strip of mesoderm cells (i.e., the notochord) lying directly beneath the ectoderm along the embryo’s midline causes a portion of the ectoderm to wrinkle and form two ridges (see figure 2). The tops of these ridges (i.e., the neural folds) curve inward and by the fourth week of gestation, meet and fuse to enclose a hollow tube (i.e., the neural tube) from which the brain and spinal cord will develop. This developmental milestone helps transform the embryo from a flattened disk to a three-dimensional primitive body. Soon after the neural folds fuse, cells originating at their junction (i.e., neural crest cells) detach and migrate to diverse locations in the embryo in order to initiate the development of many vital body structures.

As the neural crest cells migrate, increasingly complex developmental activity in the embryo results in considerable progress toward a recognizable human form. Over the course of the fourth week, the heart begins to pump; arm and leg buds appear; and the groundwork is laid for the liver, lungs, eyes, spleen, and other body systems. The embryo doubles in length to become pea size. During the next several weeks, rapid progress in development and refinement continues as nerves sprout, lymphatic and coronary vessels appear, brain structures become visible, the intestinal loop forms, the kidneys begin to ascend, and many other milestones are achieved. Hands begin to develop in the fifth week, and finger rays appear in the sixth; feet and toe rays follow a week later. In the seventh week, bone begins to replace the skeleton’s cartilage foundation, eyelids form, and the first hair follicles appear. By the end of the eighth week of gestation, the embryo is just over 1 inch long, and all major body systems are in place. From this point onward, the embryo (now termed “fetus”) primarily undergoes further refinement and growth, which continue after birth at a slower rate until adult form and stature are attained.

**Figure 2:**
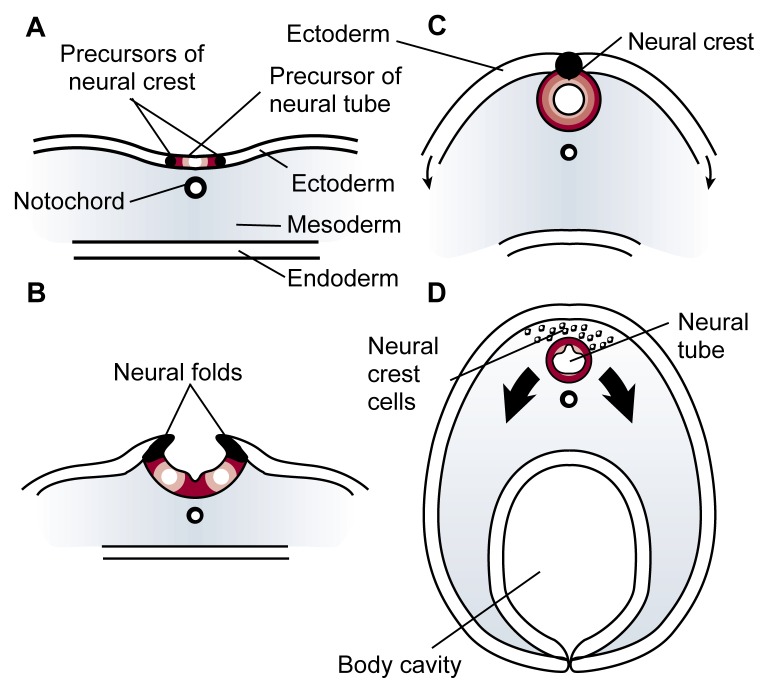
Formation of the neural tube (cross view). Early in an embryo’s development, a strip of specialized cells called the notochord (A) induces the cells of the ectoderm directly above it to become the primitive nervous system (i.e., neuroepithelium). The neuroepithelium then wrinkles and folds over (B). As the tips of the folds fuse together, a hollow tube (i.e., the neural tube) forms (C)—the precursor of the brain and spinal cord. Meanwhile, the ectoderm and endoderm continue to curve around and fuse beneath the embryo to create the body cavity, completing the transformation of the embryo from a flattened disk to a three-dimensional body. Cells originating from the fused tips of the neurectoderm (i.e., neural crest cells) migrate to various locations throughout the embryo, where they will initiate the development of diverse body structures (D).

—Mary Beth de Ribeaux

BibliographyGilbertSFDevelopmental Biology2d ed.Sunderland, MASinauer Associates1988GilbertSFRaunioAMEmbryology: Constructing the OrganismSunderland, MASinauer Associates1997LarsenWJEssentials of Human EmbryologyNew YorkChurchill Livingstone1998

*Mary Beth de Ribeaux is a science editor of* Alcohol Health & Research World.

Early diagnosis and intervention for children affected by prenatal alcohol exposure can reduce their risk for social difficulties later in life (e.g., problems with employment or trouble with the law resulting from impulsive behavior and lack of inhibition) (Streissguth et al. 1996). Identifying these children can be difficult, however. One useful FAS screening tool relies on the following trio of distinctive facial features that characterize prenatal alcohol exposure: small eye openings (i.e., short palpebral fissures), a thin upper lip, and a flattened or absent groove between the upper lip and nose (i.e., philtrum) (Astley and Clarren 1996). An excellent correlation exists between these structural malformations (i.e., dysmorphologies) and a history of prenatal alcohol exposure. Nevertheless, many children born to alcoholic mothers lack these facial defects, although the children do exhibit alcohol-related neurodevelopmental deficits, such as mental retardation, attention deficits, hyperactivity, impulsiveness, and poor judgmental skills.

To help define the risks associated with maternal alcohol use during pregnancy, researchers are seeking to understand the mechanisms of alcohol’s teratogenic effects on various tissues. The use of animal models to reproduce many of the deficits seen in FAS allows investigators to study alcohol’s teratogenic effects in detail under controlled treatment conditions. In addition, recent findings in molecular embryology have greatly advanced scientific knowledge about the signals and agents that govern normal embryo development. Applying this knowledge can help elucidate how prenatal alcohol exposure results in birth defects. It also can clarify our understanding of why certain physical defects are useful diagnostics for prenatal alcohol exposure, and it places these deformities within the overall criteria that define whether a potentially affected person may qualify for support services.

This article first reviews current understanding about the embryo’s vulnerability to facial malformations and the alcohol-induced cell death responsible for producing such deformities. The article then explores several theories on how alcohol triggers cell death in embryos and concludes with a discussion of possible future research directions.

## Alcohol-Induced Cell Death

Many toxic substances (i.e., toxicants), including alcohol, are specific in their actions and affect only a subset of cells within the embryo. The timing of exposure plays a role in the embryo’s outcome as well: A given tissue’s susceptibility to disruption peaks during distinct timeframes of development (i.e., critical sensitivity windows). Thus, a close examination of the target tissue’s developmental history may reveal clues as to the toxicant’s mechanism and the basis for the tissue’s sensitivity. Such examination also may identify additional, previously unsuspected targets. For example, because the face, limbs, urogenital tract, and central nervous system all use the same set of genes to direct their growth and development, it is not surprising that certain toxicants or genetic mutations will disrupt all of these tissues to some degree.

The concept of critical sensitivity windows is useful in examining how alcohol produces its teratogenic effects. Certain embryonic tissues, such as those of the face, heart, and urogenital tract, appear to be particularly sensitive to alcohol-induced malformations during the processes that establish location, growth, and three-dimensional shape as the tissues develop from their primitive origins. In humans, many of these early developmental events occur during a time when a woman may be unaware of her pregnancy, 3 to 6 weeks after fertilization. For example, alcohol is most likely to cause heart defects during the time when cellular signals are directing the heart’s division into its various chambers and great vessels, starting 4 weeks after fertilization.

In work that was critical in drawing attention to alcohol’s early effects on the embryo, Sulik and colleagues (1981) used a mouse model developed by Webster and colleagues (1980) to convincingly demonstrate that alcohol targets early events in organ formation. Using an experimental protocol that involved exposing mouse embryos to alcohol during a specific period of gestation (corresponding to the third week of human pregnancy),^2^ the researchers produced facial malformations consistent with the visible characteristics of FAS in people (i.e., a narrow forehead, short palpebral fissures, a small nose and midface, and a long upper lip with a deficient philtrum), as shown in figure 1. The peak maternal blood alcohol concentration (BAC) in the mice used in this research ranged from 0.2 to 0.5 percent; the lower BAC levels, in particular, are achievable in alcoholics.

The fact that alcohol causes similar facial defects in people and rodents implies that alcohol probably affects embryonic events common to both species. Closer examination of alcohol-exposed mouse embryos revealed significant cell death within specific groups (i.e., populations) of cells, including the primitive neural and facial tissues. In particular, cell death was observed in a group of cells called the neural crest (see figure 2 for similar cell death in chick embryos) (e.g., Sulik et al. 1988).

Rats exposed to alcohol during late gestation or soon after birth experienced a similar death of cells within specific brain regions. The restriction of alcohol-induced cell death to selected brain regions supports the concept that alcohol affects specific target tissues. Moreover, the causative relationship between alcohol and cell death is reinforced by the fact that several alcohol-affected embryonic tissues later result in body structures that exhibit malformations consistent with underdevelopment. In addition to the characteristic facial defects seen in FAS, these malformations can include exencephaly, a condition in which the brain is exposed or extrudes from a skull defect; spina bifida, a congenital defect in which part of the spinal cord is exposed through a gap in the backbone; absence of the corpus callosum, a brain structure that helps link the two brain hemispheres (i.e., failure of the corpus callosum to develop); and motor neuron losses in the spinal cord.

### Type of Cell Death

Both chronic and acute alcohol exposures can lead to cell death in the mature liver, brain, and certain white blood cells (i.e., lymphocytes), as observed in animal models and cell culture systems. In turn, such aberrant cell death might contribute to characteristic tissue diseases associated with repeated alcohol exposure (e.g., liver cirrhosis or immune suppression). Although some of this cell death involves necrosis, much of it is consistent with the elicitation of another type of cell death called apoptosis, in which the cell “programs” its own destruction. (For a discussion of the potential contribution of apoptosis to alcoholic liver disease, see the article by Nanji and Hiller-Sturmhöfel, pp. 325–330.)

Necrosis and apoptosis differ in several crucial ways. Necrosis occurs when cell metabolism ceases, leading to membrane disintegration and rupturing of the cell’s contents. In contrast, apoptosis depends on activation of the cell’s own internal cell-death machinery (White 1996). Apoptotic cells maintain their metabolism and membrane integrity but are characterized by damage to cellular repair systems, DNA fragmentation, and cellular breakdown into membrane-enclosed, metabolically active cell fragments (i.e., “blebs”). These distinctive characteristics are the basis for assays that differentiate apoptotic from necrotic cell death. Most cells contain apoptotic machinery but keep it checked with a series of regulatory suppressive proteins. A variety of signals may initiate apoptosis, however, including a loss of cell division controls (e.g., during the change from a normal to a precancerous cell); altered signaling from cell mediators that stimulate proliferation, differentiation, or cell survival (i.e., growth factors); changes in cell attachment to various tissue surfaces; and activation of specific proteins that trigger cell death (i.e., “death domain” proteins).

Some apoptosis is a natural part of development. In adults, for example, apoptosis is often a desirable process used to remove damaged or mutated cells (e.g., cells in tumors) that have escaped from the normal constraints on their growth. In embryos and fetuses, apoptosis shapes and remodels developing tissues by removing unwanted cell populations (e.g., the “webbing” between fingers and toes, which is deleted during the sixth through eighth weeks of gestation in humans). Research has established that the majority of cell death during *normal* embryo development is apoptotic. At critical times or locations, however, excessive cell death beyond the normal bounds of apoptosis could produce malformations within susceptible tissues, despite the embryo’s tremendous capacity to repair damage. The realization that alcohol and other teratogens can stimulate excessive cell death within the embryo was a conceptual breakthrough.

Using neuronal cells from chicks, investigators first documented embryonic cell death resulting from prenatal alcohol exposure in the late 1960’s (Sandor 1968). More than a decade later, similar studies of mouse embryos revealed clusters of dying cells, primarily in tissues that would later form the central nervous system (i.e., in the neuroepithelium) (Bannigan and Burke 1982). Apparently, the cell death observed by the researchers was apoptotic: Although the cells continued to synthesize DNA (thus indicating that they were still active), they had a rounded appearance and contained condensed material in their nucleus and surrounding cell fluid—features not inconsistent with current definitions of apoptosis. (The older scientific literature refers to embryonic cell death as necrosis, however, leading to some confusion.)

More recent studies have provided support that apoptosis is indeed the type of cell death at work in embryos exposed to alcohol. Alcohol-exposed cells within the embryo have several structural and biochemical features that are unique to apoptosis. For example, Sulik and colleagues (1988) found that cells in alcohol-exposed embryos often had nuclear DNA that was fractured and condensed into small, dense particles (i.e., pyknotic fragments) characteristic of apoptosis. Through the use of the appropriate biochemical reagents, these pyknotic fragments can be readily detected within the nervous system and neural crest of embryos exposed to alcohol (Cartwright et al. 1998). Another characteristic indicator of apoptosis is the collapse of the entire cell into small, metabolically active subunits that are soon engulfed by neighboring cells for disposal. Special dyes can penetrate these subunits, and stained subunits show up prominently during alcohol-induced cell death (Sulik et al. 1988). Further evidence that alcohol-induced cell death within the neuroepithelium and neural crest is apoptotic comes from a study showing that cell death was prevented by compounds that inhibit caspase, an enzyme that destroys intracellular repair machinery to ensure the completion of apoptosis (Cartwright et al. 1998). Taken together, these findings suggest that prenatal alcohol exposure can result in the apoptotic elimination of embryonic cells. Necrosis also can occur under certain circumstances (e.g. nutrient or oxgen starvation), however, and care must be taken to distinguish between these two mechanisms.

## Alcohol’s Effects on Neural Crest Development

Realizing that the cells of the neural crest are particularly vulnerable to alcohol-induced death, researchers have focused on this cell population. Analysis of alcohol-induced facial defects revealed certain commonalities among the affected structures. Many of these structures (i.e., the upper and lower jaw, nose, ears, orbital bone around the eye, and forehead) are derived from the same cellular ancestors in the embryo (i.e., cell lineage), a subset of the neural crest population called the cranial neural crest. This lineage originates within the primitive neural tube (i.e., the neurectoderm) (for a review, see Noden 1991). Cranial neural crest cells migrate from the neural tube and differentiate into a wide variety of structures (see table). The specific body structure that cranial neural crest cells will generate (i.e., their positional identity, such as whether the cells will contribute to the upper or lower jaw, for example) is determined before they leave the neurectoderm. Similarly, their differentiation fate (e.g., whether they develop into nerve or bone tissue) is determined before or shortly after the cells begin migration. Many of the structures arising from the cranial neural crest potentially could be affected by alcohol exposure, as a survey of the various birth defects reported in the FAS literature would show.

A separate neural crest population (i.e., the trunk neural crest) originates from the primitive spinal cord and contributes to other body structures (see table). Unlike the cranial neural crest cells, however, the fate of the trunk neural crest cells is not predetermined before their migration. Instead, the type of tissue they ultimately form depends on the environment into which they migrate. Scientific literature suggests that tissues formed from the trunk neural crest cells are less affected by alcohol than are those made by the cranial neural crest cells. Such resistance may reflect the greater adaptability the trunk neural crest cells have with respect to their identity and differentiation, even after some cells are deleted. Researchers have not formally examined this hypothesis, however.

## Alcohol and Facial Defects: Timing Is Everything

The mouse studies of Sulik and colleagues (1988) clearly highlighted the similarity between the visible characteristics of FAS and the facial malformations produced following abnormal cranial neural crest development. Widespread cell death within embryo regions enriched with neural crest cells provided strong evidence that alcohol directly targeted this cell population. Nevertheless, a key question remained: What mechanism could be triggering their death?

Recent advances in developmental biology have made this question approachable at the molecular level, because much is now known about the mechanisms involved in neural crest development. Several clues have come from research zeroing in on the role played by the precise timing of alcohol exposure in relation to the embryo’s developmental stage. Researchers have made progress in identifying the critical sensitivity window for neural crest apoptosis, when and where cell death actually occurs, and how alcohol-induced and “natural” (i.e., endogenous) apoptosis interact.

### Neural Crest Critical Sensitivity Window

To investigate the mechanism of neural crest cell death, some researchers have turned to the chick embryo, which has long been a model of choice for neural crest studies. (Despite the obvious differences among birds, mice, and people, neural crest development in each species is similar; nature does not “reinvent the wheel,” but instead uses the same genes to solve common problems of embryo development across species.) Chicks offer several advantages to investigators studying embryo development. Because the chick develops in an egg outside the mother’s body, studies can avoid potential complicating factors arising from maternal and placental metabolism and nutrition. Moreover, researchers can cut a small window in the eggshell and directly view and manipulate the embryo. Resealing the window with tape or wax provides sufficient protection to allow the chick embryo’s development to continue.

**Table:** Body Components Derived From Neural Crest Cells in the Embryo

**Structures Derived From Cranial Neural Crest Cells**
Facial cartilage and bones, including those of the nose, upper and lower jaw, and around the eye; also the small bone supporting the tongue (i.e., hyoid bone)
Skull cartilage and bone (front only)
Cartilage of the inner ear
Tooth core (i.e., odontoblasts)
Connective tissue of the thymus and the thyroid, salivary, pituitary, and tear-producing (i.e., lacrimal) glands
Calcitonin-producing cells of the thyroid gland
Transparent fibrous supportive tissue (i.e., stroma) and inner lining of the cornea
Connective tissue surrounding the eye and optic nerve
Eye muscles that control the size of the pupil and curvature of the lens (i.e., pupillary and ciliary muscles)
Skin of the face and front part of the neck, including the inner, living layer of skin (i.e., dermis); smooth muscle; and fat cells
Pigment-producing skin cells (i.e., melanocytes)
Sensory nerve portions of several nerves arising directly from the brain, specifically cranial nerves III (oculomotor), V (trigeminal), VII (facial), IX (glossopharyngeal), and X (vagus)
Specialized supportive and nutritive tissue (i.e., glial cells) of all cranial nerve cell bundles (i.e., ganglia)
Innermost and middle membrane layers (i.e., the pia mater and arachnoid mater, respectively) that enclose the lower rear (i.e., occipital) region of the brain
Smooth muscle and dividing walls (i.e., septa) of the pulmonary artery and aorta (i.e., cardiac neural crest)
Type I cells of the small body structure involved in monitoring blood levels of oxygen, carbon dioxide, and hydrogen (i.e., the carotid body)
**Structures Derived From Trunk Neural Crest Cells**
Motor neurons of the involuntary (i.e., sympathetic and parasympathetic) portion of the nervous system outside of the brain and spinal cord (i.e., the peripheral nervous system)
Intestinal, preaortic, and spinal nerve cell bundles (i.e., ganglia)
Specialized supportive and nutritive tissue (i.e., glial cells) of the peripheral nervous system
Sheath-producing cells in the peripheral nervous system (i.e., Schwann cells)
Innermost and middle membrane layers (i.e., the pia mater and arachnoid mater, respectively) enclosing the spinal cord
Epinephrine- and norepinephrine-releasing cells in the adrenal medulla (i.e., chromaffin cells)
Pigment-producing skin cells (i.e., melanocytes)

SOURCES: Noden, D.M. Vertebrate craniofacial development: The relation between ontogenetic process and morphological outcome. *Brain, Behavior and Evolution* 38(4–5):190–225, 1991; Larsen, W.J. *Human Embryology*. New York: Churchill Livingstone, 1993; Hall, B.K., and Hörstadius, S. *The Neural Crest*. London: Oxford Science Publications, 1988.

As with mouse embryos, chick embryos exposed to alcohol levels greater than 0.15 percent exhibit significant cell death within regions enriched with cranial neural crest cells and distinct sections of the primitive brain (i.e., the midbrain and hindbrain neural folds) (Cartwright et al. 1998). Using specific markers, researchers documented directly that neural crest populations are deleted by alcohol exposure, and a laboratory technique (i.e., double-stained overlays) confirmed that these losses are attributable to cell death. In addition, just as in mouse embryos, comparatively little cell death occurs in the heart, eyes, and precursors of the vertebral column and skeletal muscle (i.e., somites) of the chick embryos. This observation suggests that cell death is not likely to be a consequence of generalized toxicity, but rather that particular embryonic cell populations are susceptible (or resistant) to this alcohol-induced effect.

Studies using mouse embryos pinpointed the critical sensitivity window for facial defects to the short time during the creation of the embryo’s three basic cell layers (i.e., gastrulation) and the first organization of the primitive nervous system (i.e., neurulation and early somite development) (Webster et al. 1980). Researchers also found the identical critical sensitivity window in chicks (Cartwright and Smith 1995).

The ability to precisely identify the developmental stage of chick embryos allowed even more refined investigation of the window’s extent with further studies. These studies uncovered a curious finding about alcohol-induced cell death in chick embryos: Alcohol exposure caused cell death only if the alcohol was administered before the neural crest cells emigrated from their birthplace in the neurectoderm. Once the cells began migrating toward the site where the chick’s face would develop, the neural crest cells were resistant to alcohol-induced death; moreover, alcohol exposure after the start of neural crest cell migration no longer resulted in facial defects. Thus, at least for chicks, the critical sensitivity window for producing facial malformations is extremely narrow, lasting only from gastrulation to cranial neural crest migration, or from 18 to 36 hours of egg incubation after laying.

Determining the critical sensitivity window for facial malformations in people is not possible, but because mice and chicks represent such diverse species sharing a similarly narrow window, these findings strongly suggest that a comparable window exists for humans. If so, then the appearance of facial defects in FAS may reflect peak blood alcohol concentration in early gestation more strongly than previously suspected.

### Dependence on Developmental Stage

Recent studies demonstrate that the regulation of apoptosis depends on the embryo’s developmental stage. In chick embryos, initial alcohol exposure and peak alcohol concentration achieved any time before neural crest cell migration do not actually result in cell death until later, specifically at 46 to 48 hours of incubation (Cartwright et al. 1998). Thus, alcohol exposure during a variety of early stages appears to activate the apoptotic pathway, but cells do not carry out the “self-destruct” sequence until they reach a particular stage of development.

Closer examination of where apoptosis is occurring in the embryo at a given timepoint further supports this theory. Because embryos develop in a head-to-tail (i.e., rostrocaudal) sequence, the neural folds do not meet along their entire length simultaneously, but are “zipped” together to form the neural tube. Thus, the neural crest cells arising from the foremost part of the neural tube are created and mature first, whereas those cells toward the opposite end of the tube are progressively less mature at any given timepoint. Likewise, exposure to alcohol at an early point in embryo development produces maximal cell death in the more rostral neural crest cell populations (e.g., the midbrain portion of the neural tube), whereas later exposure shifts the majority of cell death to more caudal populations (e.g., the hindbrain segment of the neural tube) (Cartwright and Smith 1995). This finding suggests that neural crest cells must achieve a particular maturation state before carrying out their alcohol-induced “suicide” and implies the existence of certain cellular events that convey susceptibility (or resistance) to apoptosis.

### Alcohol-Induced and “Natural” Apoptosis

**Figure 3:**
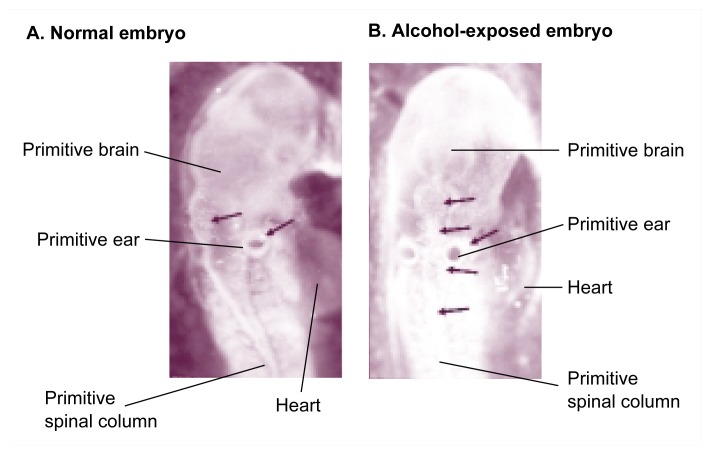
Apoptosis in neural crest cells and the primitive brain. Panel A shows a normal 48-hour embryo stained with a bright dye (i.e., acridine orange) that becomes concentrated in apoptotic cells. These dead cells appear as tiny dots (see arrows). Panel B shows a 48-hour embryo that was exposed to alcohol at a critical developmental time (i.e., at 18 to 36 hours of incubation). Many more dead cells, visible as bright dots (see arrows), are seen in the primitive brain and face of the alcohol-exposed embryo compared with the normal embryo. Note that some cell groups are dying in both embryos, but many more cells are dying in the alcohol-exposed embryo. This observation supports the premise that normal and alcohol-induced cell death occur simultaneously in the early brain and neural crest.

Mouse studies by Sulik and colleagues (1988) provided another significant clue regarding apoptosis regulation. Their research found that teratogen-induced cell death often was most common in embryonic regions already undergoing some degree of endogenous cell death. Cartwright and Smith (1995) noted the same observation in the chick model, in which alcohol-induced neural crest apoptosis coincided with the normal deletion of two subgroups of cranial neural crest cells by endogenous apoptosis (see figure 3).

The coincidence of alcohol-induced and normal neural crest death prompted the hypothesis that alcohol exposure aberrantly activates the apoptotic machinery in cranial neural crest cells. This hypothesis predicts that the prevalence or activity of substances involved in endogenous apoptosis would increase during concurrent alcohol-induced death. Molecular analysis did not support this prediction, however: A study by Cartwright and colleagues (1998) found no increase in the prevalence or activity of two substances involved in endogenous apoptosis (i.e., a transcription factor named msx2 and a growth factor named BMP-4) (Cartwright et al. 1998) during alcohol-induced death. Thus, although the timing of alcohol-induced and endogenous apoptosis coincides, their processes must be activated through different pathways. This finding opens the possibility of discovering therapies that could inhibit alcohol’s actions without altering the necessary endogenous cell deletions.

## What Triggers Alcohol-Induced Apoptosis?

Understanding the molecular regulation of apoptosis will help explain how alcohol initiates the apoptotic pathway and results in the death of some, but not all, cell populations. In pursuit of this goal, current research efforts focus on identifying the mechanism that activates the cell-death pathway following alcohol exposure. In all likelihood, the trigger for alcohol-induced apoptosis lies within the target cell population itself, because many other cell populations do not apoptose, despite equivalent alcohol exposures. Investigators have presented several hypotheses to explain alcohol’s activation of apoptosis, each with strong supporting evidence. The following sections explore these hypotheses and the evidence to support them.

### Retinoic Acid Deficiency

Retinoic acid (RA), an active form of vitamin A, is essential for neural crest survival and is a key regulator of body form and structure development (i.e., morphogenesis). RA affects the positional identity of cells according to the structure that they will form within diverse embryonic regions, including the cranial neural crest, face, central nervous system, limbs, and urogenital tract. In addition, RA is a potent mediator of differentiation in many cell lineages. Alcohol exposure is associated with localized RA deficiencies in embryos, however (DeJonge and Zachman 1995; Deltour et al. 1996). Because low levels of RA result in the apoptosis of neural crest populations and their subsequent elimination from the embryo (Maden et al. 1997), RA deficiency is implicated in alcohol-induced apoptosis.

Studies of a class IV alcohol dehydrogenase enzyme found in embryos further supports this hypothesis. This enzyme is involved in the synthesis of retinal, an immediate precursor of RA. Deltour and colleagues (1996) demonstrated that alcohol concentrations found in alcoholic women inhibit this enzyme and are associated with reduced RA levels within the embryonic face and nervous system. Additional research shows that treatment with supplemental RA prevents alcohol-induced heart defects in embryos (Twal and Zile 1997).

Although frank deficiency of vitamin A compounds (i.e., retinoids) is seldom observed in Western populations, a recent study in Iowa (Duitsman et al. 1995) found that between 9 and 26 percent of a sample of low-income pregnant women in their third trimester had marginal retinoid stores, equivalent to levels seen in malnourished, non-industrialized populations. Inadequate vitamin A intake among alcoholic women could create marginal retinoid concentrations within the embryo, possibly increasing the risk for RA-and alcohol-related birth defects. Paradoxically, however, RA itself is also a potent teratogen, and excessive RA or vitamin A supplementation could endanger the embryo. Scientists need better documentation of potential alcohol-RA links before contemplating nutritional interventions.

### Free Radical Damage

An alternative hypothesis on the mechanism behind neural crest apoptosis is based on alcohol’s known ability to cause cell injury during its metabolism. Alcohol exposure can increase the levels of highly reactive oxygen molecules known as free radicals as well as deplete cells of the protective compounds (i.e., antioxidants) that normally scavenge these toxic molecules. Under such conditions (i.e., oxidative stress), vital components of the cell may begin to combine with free radicals and result in injury. In particular, free radicals can damage cell membranes through a process called lipid peroxidation. This process may interfere with many important cell regulatory processes, including control over substances entering and leaving the cell, intercellular communication, and protein synthesis. Damage from such interference could be fatal to cells.

Approaches using whole embryo and cell cultures, particularly the research conducted by Chen and colleagues (Chen and Sulik 1996; Chen et al. 1996) have been critical in testing the hypothesis relating free radical damage to neural crest apoptosis. Noting that cultured neural crest cells appear to lack an antioxidant compound (i.e., superoxide dismutase) that plays an important role in removing free radicals, the researchers cultured alcohol-exposed neural crest cells together with superoxide dismutase and other free radical scavengers. The results showed that the addition of antioxidants modestly reduced alcohol-induced cell death (Chen and Sulik 1996). Because free radical mechanisms also participate in endogenous apoptosis, however, it is unclear whether antioxidants interfere Cell Death in the Embryo with alcohol’s action or with the apoptosis process itself.

Supplementation with gangliosides, a type of lipid compound usually found abundantly in nerve cell membranes, also decreases the death of alcohol-exposed neural crest cells (Chen et al. 1996), and protocols similar to those used with antioxidant supplementation have protected nerve cells exposed to alcohol. Thus, researchers hypothesize that gangliosides may defend against cell membrane damage from lipid peroxidation.

Further research will prove informative in understanding free radical damage and how alcohol-induced oxidative stress contributes to alcohol’s injury to the embryo. Alcohol exposure may result in free radical production from several sources. For example, free radicals could be formed as a consequence of alcohol’s oxidation itself (e.g., through enzymes, such as cytochrome P450’s, or catalases); its alteration of the cell’s potential for chemical reactions or the chemical chain of events involved in generating cell energy (i.e., mitochondrial respiration); or the action of other agents, such as nitric oxide. The sensitive intracellular probes that are now available for detecting free radicals and their sources will enable researchers to test these mechanisms directly.

### Intracellular Communication Interference

A third mechanism proposed for alcohol-induced apoptosis involves alcohol’s ability to perturb the function of cell membrane-associated proteins. These proteins function as channels or receptors that transfer critical chemical signals from the cell surface to its interior (i.e., transmembrane or intracellular communication). Interference with this process, perhaps through direct alcohol-protein interactions, could cause aberrant activation or inhibition of the communication pathways vital to cell survival.

Studies using various cultured cell lines have documented alcohol’s ability to alter intracellular communication through a variety of mechanisms (e.g., alterations of cyclic AMP, intracellular calcium release, and activity of the enzyme protein kinase), depending on the target cell (Slater et al. 1993; Stachecki et al. 1994). Thus, one can easily speculate that alcohol could interfere with the signaling of a growth factor critical to a cell’s survival at particular timepoints during development and lead to apoptosis. The fact that a wider range of embryonic tissues is not overtly affected by alcohol exposure may be attributed to the embryo’s tremendous capacity to repair damage. In addition, the redundancy of cellular signals may help compensate for alcohol’s interference. The power of such compensation can be seen most clearly in studies that delete or inactivate a critical gene, yet often result in normal embryos.

Again, recent advances in deciphering the molecular signals that govern development will greatly facilitate further exploration of altered cellular communication as a potential mechanism of apoptosis. Quite likely, a combination of RA deficiency, free radical damage, and interference with cellular communication, as well as other mechanisms, all contribute to alcohol’s toxicity and subsequent pathologies.

## Future Directions

Advances in molecular and cellular biology have opened multiple avenues for exploring alcohol’s impact on prenatal development. Many of alcohol’s target tissues, including face, nervous system, limb, and urogenital tract tissues, employ a common set of genes that are responsible for coordinating tissue growth (Johnson and Tabin 1997). Several alcohol-related birth defects are consistent with altered embryo development, and examining the role that genes play in regulating early development in animal models may yield insights into how susceptible tissues respond to alcohol exposure. In particular, the use of mice with deliberate gene mutations inserted in their genetic material (i.e., transgenic mice) will assist investigators seeking to connect specific gene alterations to alcohol’s developmental consequences. (See article by Homanics and Hiller-Stumhöfel, pp. 298–309.)

New approaches also are available to study alcohol’s effects on the cell’s normal metabolic processes and communication. For example, highly sensitive intracellular reagents and probes allow scientists to monitor the various metabolic pathways that link events in the cell membrane to changes within the cell. Combining these techniques with specialized computer-driven detection equipment, such as laser-driven microscopy (i.e., confocal microscopy), will enable investigators to monitor alcohol’s short-term effects on single cells as they occur.

Finally, although the cranial neural crest contributes to many tissues, alcohol-related research on these cells predominantly focuses on facial bone and cartilage. Examination of other tissues arising from the cranial neural crest could uncover less recognized targets of prenatal alcohol exposure. Approaches similar to those undertaken by researchers studying facial defects also will benefit studies of brain, heart, urogenital tract, and limb development.

Taken together, recent discoveries in cellular and molecular embryology will greatly advance our understanding of alcohol’s impact on prenatal development. Moreover, understanding the limited circumstances that create alcohol-induced facial defects may bolster the recognition that children who demonstrate alcohol-related neurodevelopmental deficits but lack overt facial defects represent a syndrome that can equal FAS in severity and long-term prognosis.

## Figures and Tables

**Figure 1:**
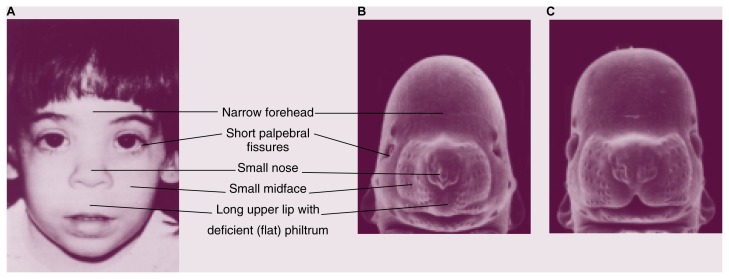
Similarities of facial defects found in (A) humans and (B) mice exposed prenatally to alcohol. Panel C shows a control mouse fetus not exposed to alcohol. *(Photograph courtesy of Kathy K. Sulik.)*

**Figure 2:**
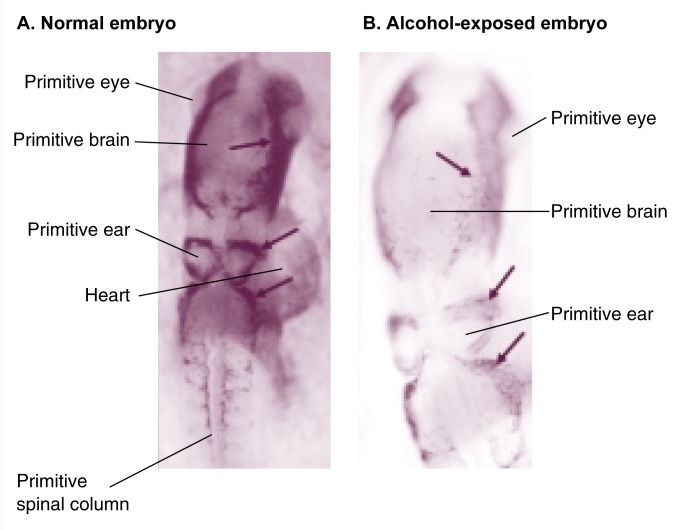
Neural crest cell death in the chick embryo. Panel A shows a normal embryo after 48 hours of incubation (corresponding to 22 to 25 days of human gestation), when the dark-stained neural crest cells (see arrows) migrate from the primitive brain toward regions of facial development. Panel B shows a 48-hour embryo that was exposed to alcohol at a critical developmental time (i.e., at 18 to 36 hours of incubation). The diffuse, weblike staining (see arrows) in the alcohol-exposed embryo indicates the presence of fewer neural crest cells compared with the many neural crest cells indicated by the denser staining in the control embryo.

## Footnotes

1 For a definition of this and other technical terms used in this article, see glossary, p. 295.

2 Protocols with alcohol exposure during other specific periods of gestation produced malformations in different body systems (e.g., the heart and limbs), as predicted by the critical windows hypothesis.

1 Although embryo development is basically the same in all mammals, the timeframes given here correspond specifically to humans.

## Glossary

Dysmorphology: Abnormal development of tissue form and structure; a congenital malformation or birth defect.
Ectoderm: The outermost of the three distinct layers of cells in the early embryo—enclosing the *mesoderm* and *endoderm—*from which the nervous system and skin ultimately form.
Endoderm: The innermost of the three distinct layers of the early embryo. The endoderm produces the lining of the digestive tract and its associated organs (i.e., the pancreas, liver, and so forth).
Gastrulation: Extensive cell rearrangements that result in three distinct cell regions (i.e., germ layers) within the embryo: the *ectoderm, mesoderm*, and *endoderm*. All subsequent embryo tissue is derived from one of these three germ layers. In humans, gastrulation begins to take place approximately 16 days after fertilization.
Mesoderm: The middle layer of the three distinct layers of the early embryo—lying between the *ectoderm* and *endoderm—*from which several of the body’s organs (i.e., the heart, kidneys, and gonads), bones and musculature, and blood cells ultimately form.
Morphogenesis: The development of the form and structure of the body and its parts, including the growth and differentiation of cells and tissues.
Neural crest: Cells at the junction of the folded tips that enclose the *neural tube*. In humans, these cells detach from the neural tube during the fourth week of gestation and migrate through the embryo to initiate development of a wide range of body structures (see table p. 291).
Neural tube: The embryological structure from which the brain and spinal cord develop. The neural tube is formed when the *mesoderm* stimulates the midline of the embryo *ectoderm* to fold up and over, transforming the embryo’s shape from a flattened disk to a primitive vertebrate body.
Neurectoderm: The primitive *neural tube*.
Somite: Any of the paired segmented divisions of the *mesoderm* that develop along the *neural tube*, forming the vertebral column. The somites differentiate into bones, connective tissue, voluntary muscle, and the deeper layers of the skin.
Teratogen: Any agent capable of causing abnormalities during prenatal development.
